# Supplementary material for: Course of post COVID-19 disease symptoms over time in the ComPaRe long COVID prospective e-cohort
Source: Nat Commun. 2022 Apr 5;13:1812. doi: 10.1038/s41467-022-29513-z (PMC8983754; doi:10.1038/s41467-022-29513-z)

Supplementary materials for “Course of post COVID-19 disease symptoms over time in the ComPaRe long COVID prospective e-cohort”

Supplementary material 1: Follow-up of patients included in the study. As data collection is in progress, all figures may improve with time. *Unavailable data may be due to 1) patients who have not yet reached the time point; 2) who reached the time point less than 30 days ago and may still answer the online questionnaire; and 3) patients actually lost to follow-up. Only the latter can cause informative censoring and bias. For other patients, we can assume that their survival prospects are similar to those of the participants who continue to be followed.

	Number of patients enrolled in the cohort	Number (%) of patients With available data*	Number(%) of patients with actual missing data at the observation point
At least 2 months since enrollment (enrolled before September 2021)	968	789 (81.5)	168 (17.4)
At least 4 months since enrollment (enrolled before July 2021)	924	711 (76.9)	167 (18.1)
At least 6 months since enrollment (enrolled before May 2021)	766	551(71.9)	139 (18.1)
At least 8 months since enrollment (enrolled before March 2021)	576	329 (57.1)	74 (12.8)
At least 10 months since enrollment (enrolled before January 2021)	250	120 (48)	0

Supplementary material 2: Day-by-day trends in the prevalence of post COVID-19 disease symptoms (A) and in their impact on patients' lives (B) (n=968 [raw data]) A: The figure presents the day-by-day prevalence of each of the 53 symptoms assessed by the Long COVID ST (grey lines). Examples of specific symptoms have been highlighted (coloured lines). For each symptom and at each observation point, we assumed that patients were either "experiencing" or "not experiencing" the symptom. We assumed that their state at an arbitrary time was the same as the state at their previous observation point and that their states before their first observation and after their last observation are unknown. B: The figure presents the day-by-day evolution of 6 domains of patients' lives that can be affected by post COVID-19 disease (assessed by the Long COVID IT). For each item and at each observation point, we modelled patients answers as either "reporting" a significant impact of the disease on this domain" (i.e., item score >7) or "not reporting" this impact (i.e., item score <8). We assumed that their state at an arbitrary time was the same as the state at their previous observation point and that their states before their first observation and after their last observation are unknown. The red lines represent a similar model for the Patient Acceptable Symptomatic State (PASS) of the long COVID IT, which is the long COVID IT score below which 75% of patients find that their disease state is acceptable.

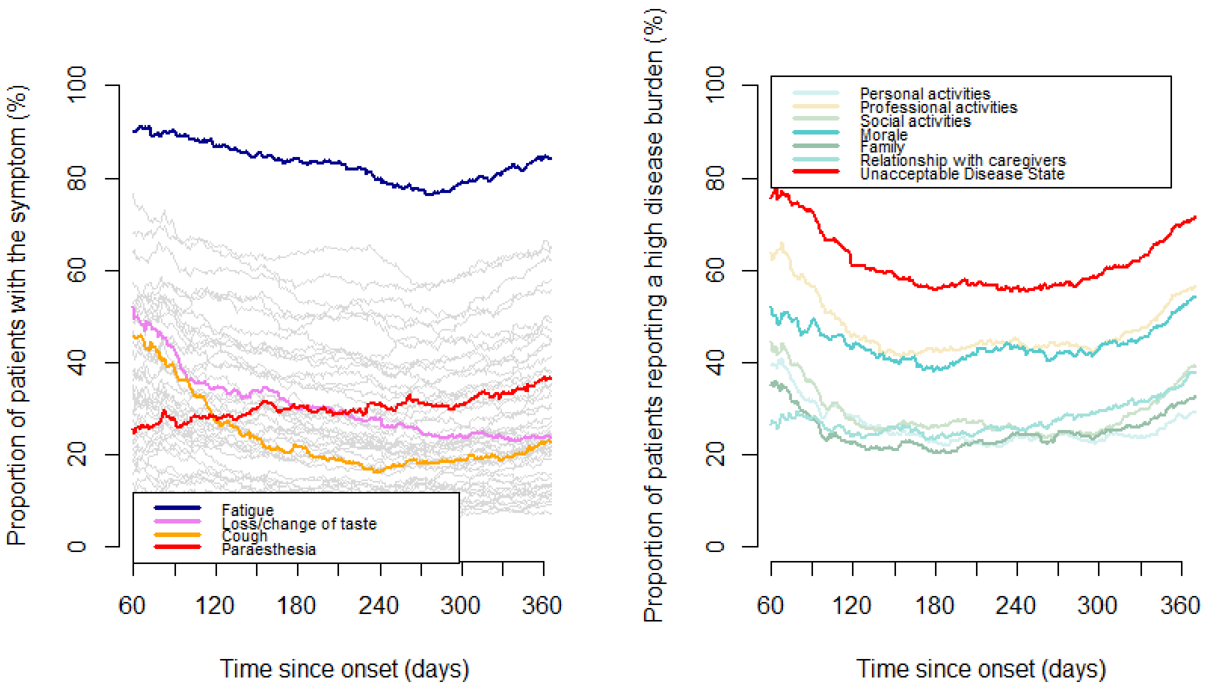

Supplementary material 3: Estimated prevalence of each of the 53 symptoms assessed by the Long COVID ST at 60 and 360 days after disease onset (n=968 [weighed data]). Prevalence is estimated by a multistate model where patients were considered to be either "experiencing" or "not experiencing" each symptom. On the assumption that a patient's state at an arbitrary time was the same as the state at their previous observation time and that each patient's states before the first observation and after the last observation are unknown, we estimated the prevalence of the given symptom day by day from disease onset. Colours indicate symptoms with a prevalence decreasing over time (green), symptoms with no change in prevalence over time (yellow), and symptoms with an increasing prevalence over time (red).

Symptom	Prevalence at 60 days	Prevalence at 360 days	Difference
Loss of appetite	30.6	6.9	-23.7
Change/Loss of taste	46.9	24.1	-22.8
Cough	44.6	21.8	-22.8
Headache	82.8	63.7	-19.1
Fever and chills	41	22.9	-18.1
Change/Loss of smell	46.9	29	-17.9
Diarrhoea	38.7	22.9	-15.8
Changing mood/impact on morale	61.1	46.2	-14.9
Sore tongue/mouth, dysphagia	33.1	18.3	-14.8
Dry skin/peeling	35.3	21.3	-14
Muscle aches	54.6	42.2	-12.4
Fatigue	93	81	-12
Dizziness/malaise	55.2	43.3	-11.9
Brain fog/Difficulty concentrating	71.8	60.2	-11.6
Balance disorder	33.8	22.7	-11.1
Nausea/Vomiting	27.6	16.7	-10.9
Sleeping more	32.5	21.7	-10.8
Tachycardia/palpitations/Arrhythmia	43.8	33	-10.8
Chest pressure	44.1	33.7	-10.4
Tremor	23.1	13.6	-9.5
Difficulty sleeping	70.1	60.8	-9.3
Swollen lymph nodes	14.8	6.2	-8.6
Skin rash	28	20.1	-7.9
Abdominal pain	41.9	34.4	-7.5
Sharp sudden pain, chest burns	32.1	24.8	-7.3
Memory problems	55.7	50.2	-5.5
Body aches	43.6	38.3	-5.3
Costal pain	29.9	25.8	-4.1
Weight loss	12	7.9	-4.1
High or low blood pressure	15.3	11.5	-3.8
Tinnitus	38.9	36.1	-2.8
Sweats	20.5	18.3	-2.2
Hot Flushes	22.3	20.7	-1.6
Congested/Runny nose	33.9	34.1	0.2
Dyspnoea	44.2	44.5	0.3
Clogged ears	11.1	11.5	0.4
Word-finding problems	47.5	48.3	0.8
Gynaecological problems	5.2	6.3	1.1
Phono/photophobia	10.8	12.7	1.9
Circulatory problems (incl. Bulging veins)	6.1	8.3	2.2
Spontaneous bruises	5.7	8.3	2.6
Heat/Cold intolerance	9.2	11.8	2.6
Hypoesthesia	3.2	5.9	2.7
Dry eyes	23.9	28.4	4.5
Urinary symptoms	8.5	13.4	4.9
Discolouration/swelling of hands and feet	6.2	11.3	5.1
Heavy legs/swelling of the legs	14.3	19.8	5.5
Blurry vision	26.2	32	5.8
Hair loss	8.2	15.1	6.9
Bone and Joint Pain	32.2	41.4	9.2
Ear pain	5.2	16.9	11.7
Paraesthesia	21.1	33.7	12.6
Neck, back and low back pain	23.4	38.5	15.1

Supplementary material 4: Estimated prevalence of symptoms of male and female patients (n=968 [weighted data]). For each of the 53 symptoms assessed by the long COVID ST, the day-by-day prevalence in male and female patients was calculated (all lines). Some symptoms are highlighted (coloured lines).

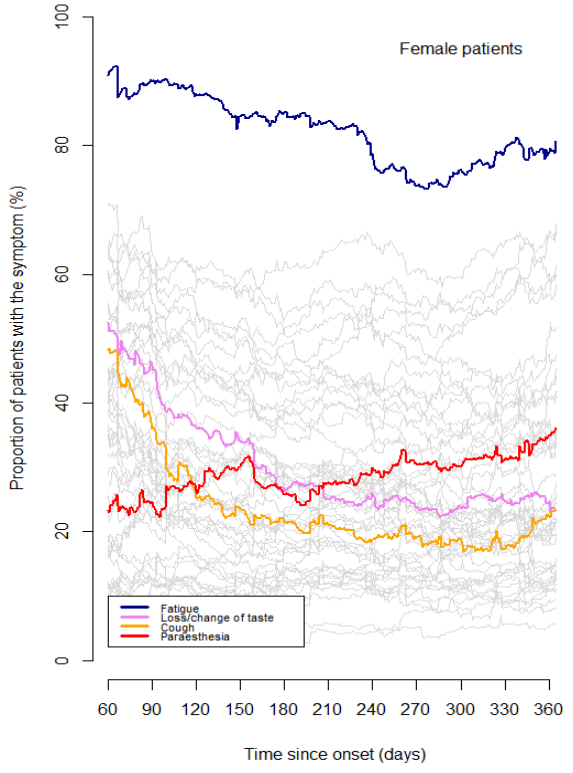

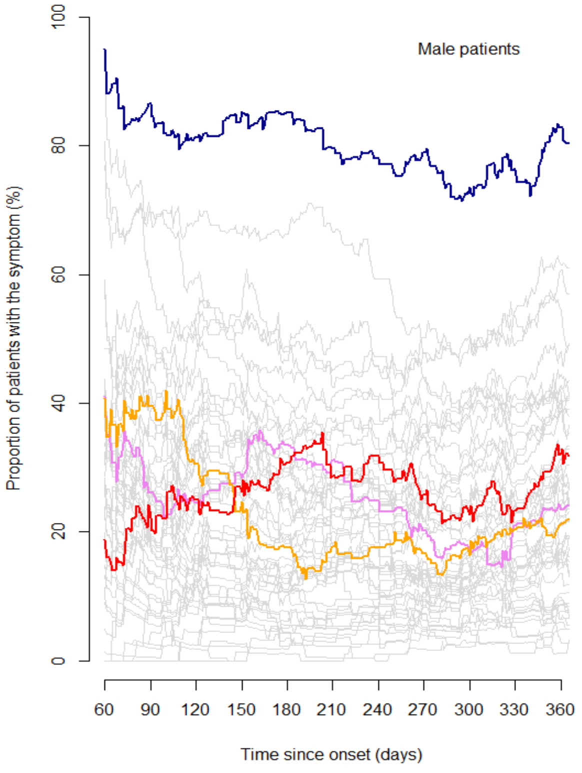

Supplementary material 5: Estimated prevalence of symptoms of patients with and without comorbidities (n=968 [weighted data]). For each of the 53 symptoms assessed by the long COVID ST, the day-by-day prevalence in patients with and without comorbidities was calculated (all lines). Some symptoms are highlighted (coloured lines).

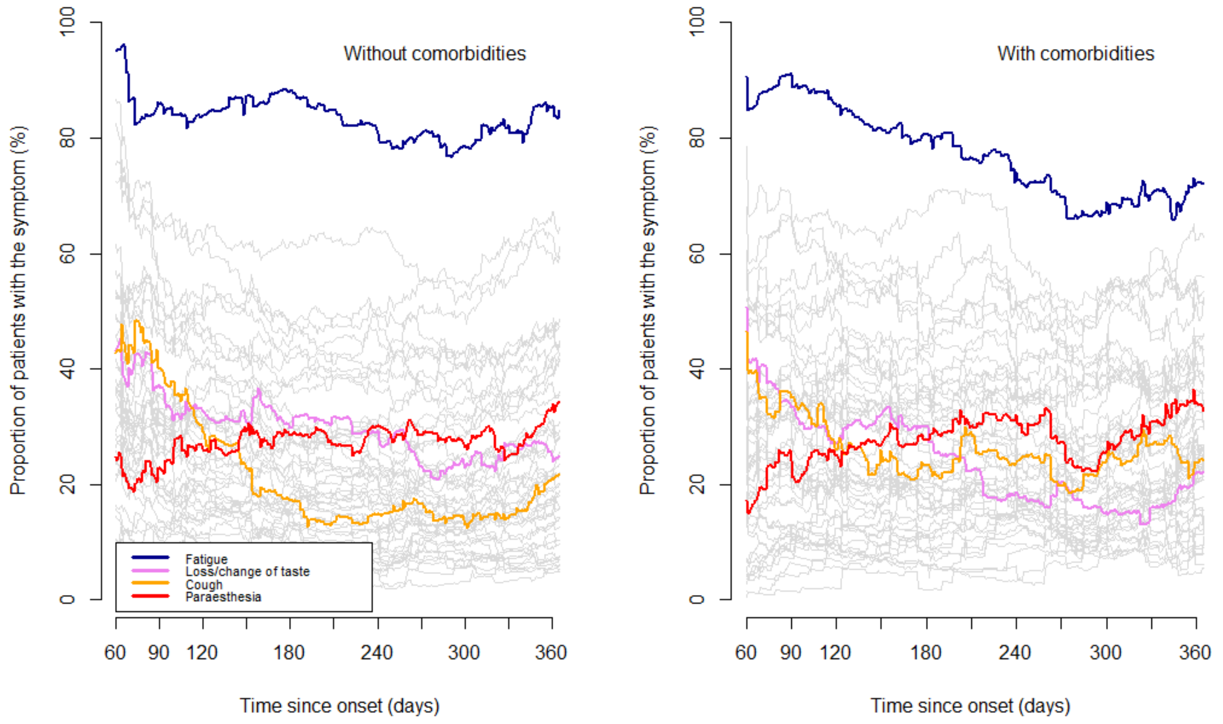

Supplementary material 6: Estimated prevalence of symptoms of aged ≤ 40 years and of those >40 years (n=968 [weighted data]). For each of the 53 symptoms assessed by the long COVID ST, the day-by-day prevalence in patients aged 40 years or less, and those older than 40 years was calculated. We present below the 6 symptoms for which the sum of absolute differences in prevalence between the two subgroups from 60 to 360 days exceeds 10%).

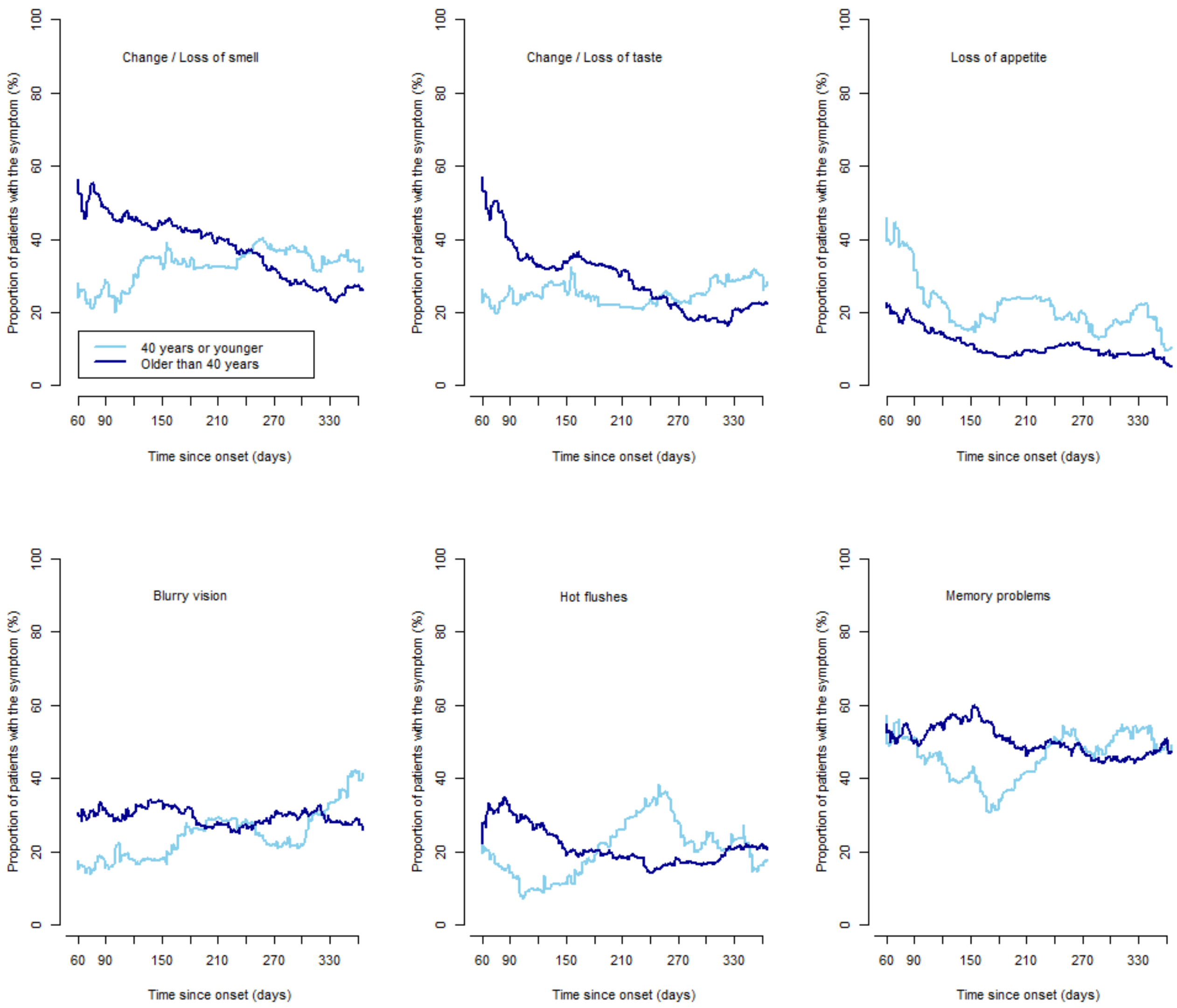

Supplementary material 7: Longitudinal course of the frequency of symptoms for patients with post COVID-19 disease (n=968 [weighted data]). Data come from the answer to the question: "how often did your symptoms occur during the last 30 days," with four response options: "permanent symptoms", "daily relapses", "weekly relapses", and "less than weekly relapses."

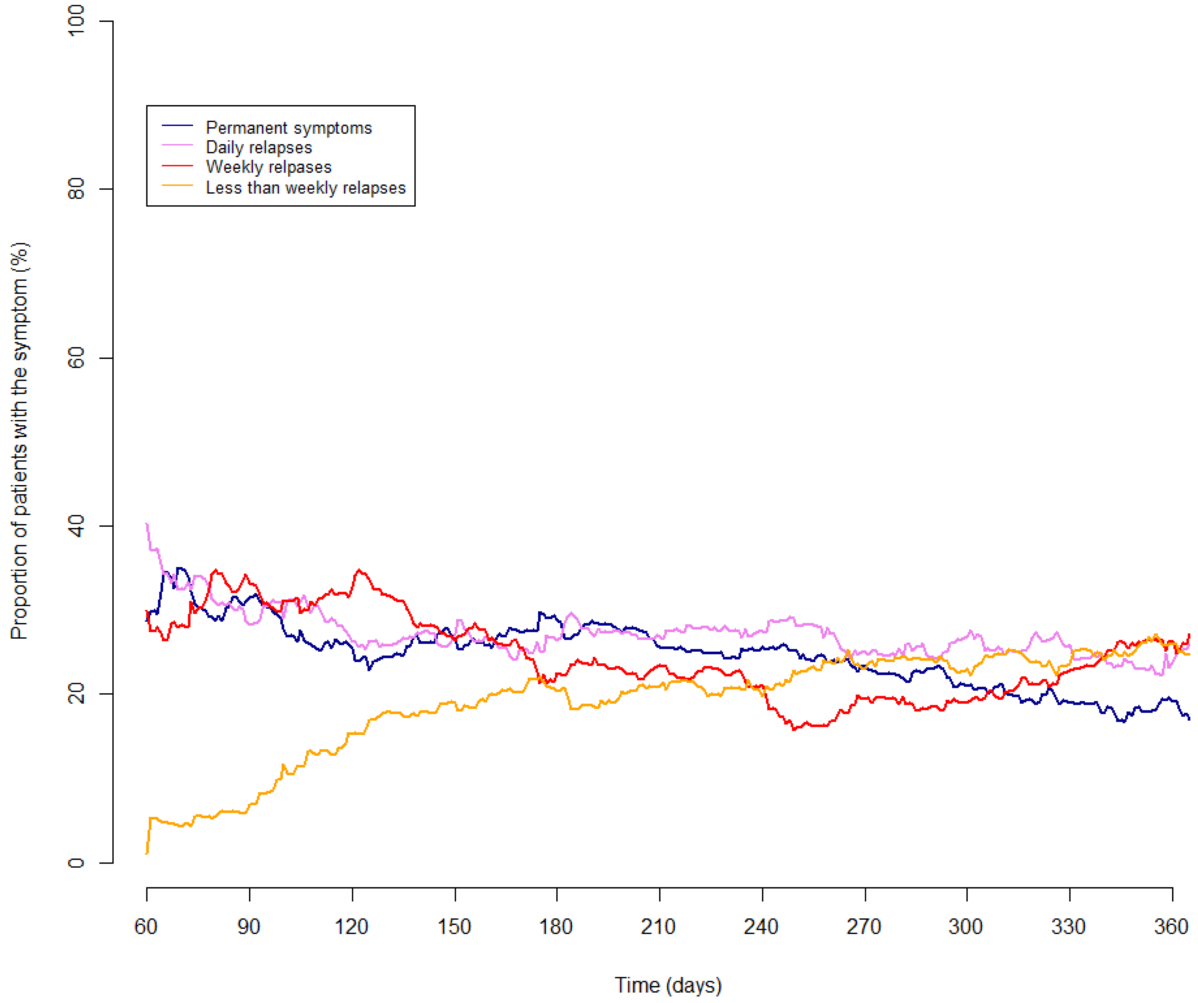

Supplementary material 8: Evolution of patients' perception of the impact of post COVID-19 disease on their lives for male and female patients (n=968 [weighted data]). The figure presents the day-by-day evolution of 6 domains of patients' lives that can be affected by post COVID-19 disease (assessed by the Long COVID IT). For each item and at each observation point, we modelled patients answers as either "reporting" a significant impact of the disease on this domain" (i.e., item score >7) or "not reporting" this impact (i.e., item score <8). We assumed that their state at an arbitrary time was the same as the state at their previous observation point and that their states before their first observation and after their last observation are unknown. The red lines represent a similar model for the Patient Acceptable Symptomatic State (PASS) of the long COVID IT, which is the long COVID IT score below which 75% of patients find that their disease state is acceptable.

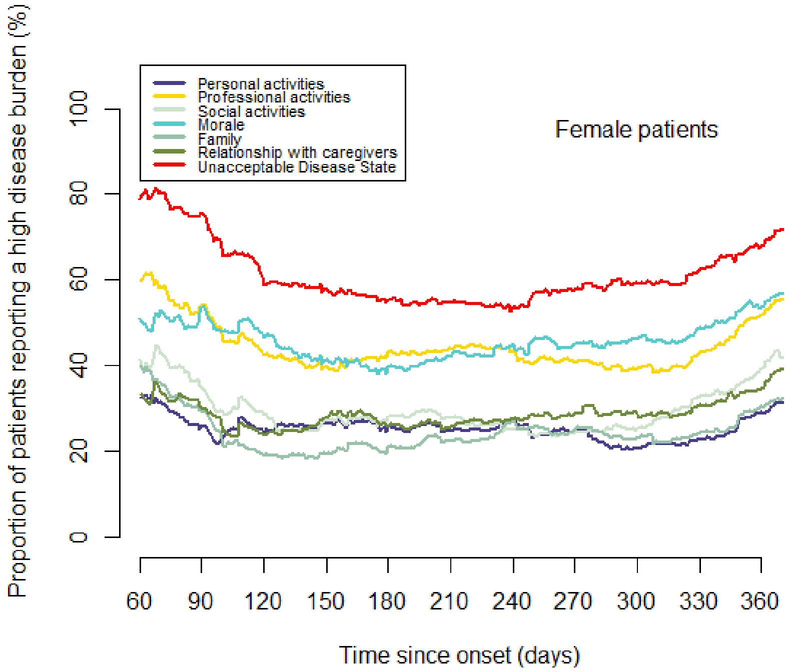

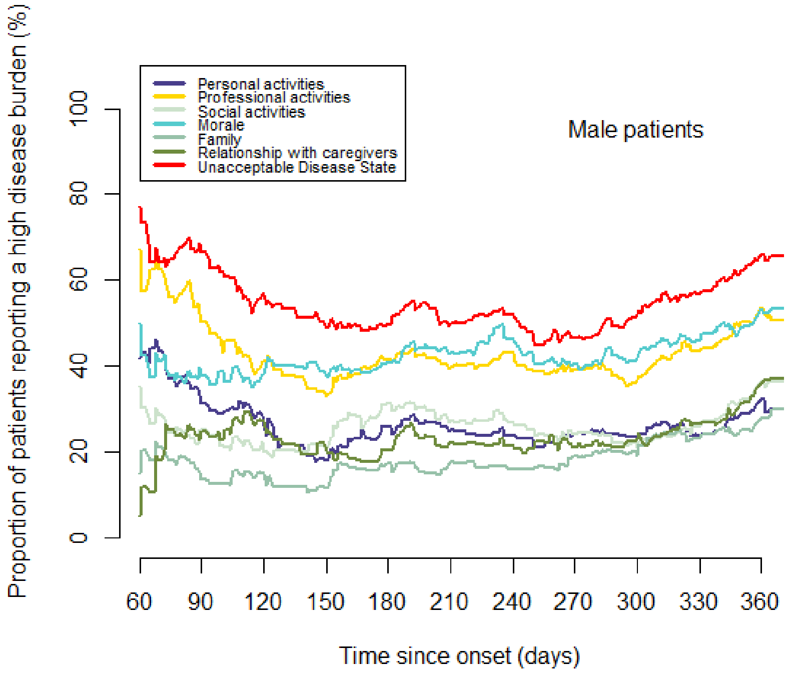

Supplementary material 9: Evolution of patients' perception of the impact of post COVID-19 disease on their lives between patients aged ≤ and > 40 years (n=968 [weighted data]). The figure presents the day-by-day evolution of 6 domains of patients' lives that can be affected by post COVID-19 disease (assessed by the Long COVID IT). For each item and at each observation point, we modelled patients answers as either "reporting" a significant impact of the disease on this domain" (i.e., item score >7) or "not reporting" this impact (i.e., item score <8). We assumed that their state at an arbitrary time was the same as the state at their previous observation point and that their states before their first observation and after their last observation are unknown. The red lines represent a similar model for the Patient Acceptable Symptomatic State (PASS) of the long COVID IT, which is the long COVID IT score below which 75% of patients find that their disease state is acceptable.

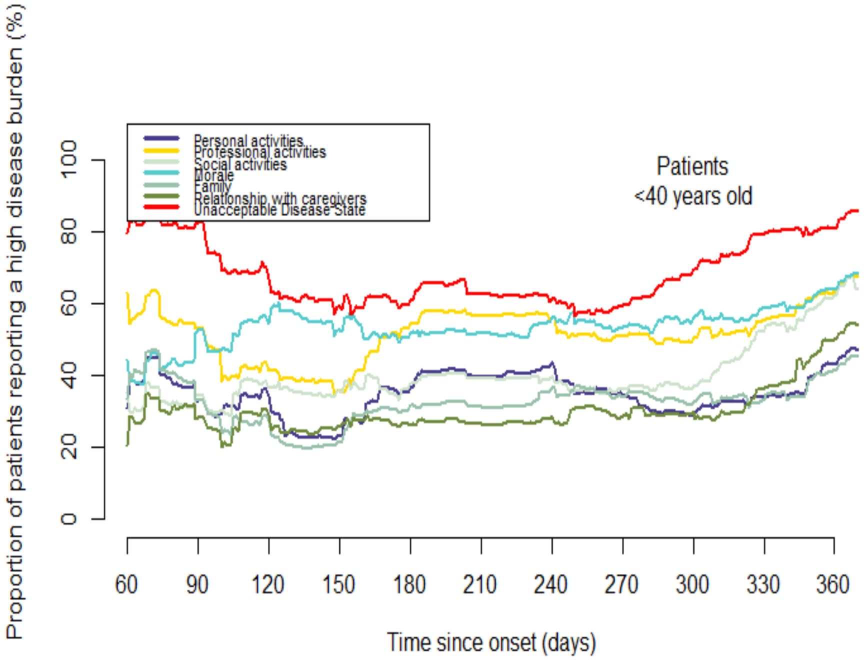

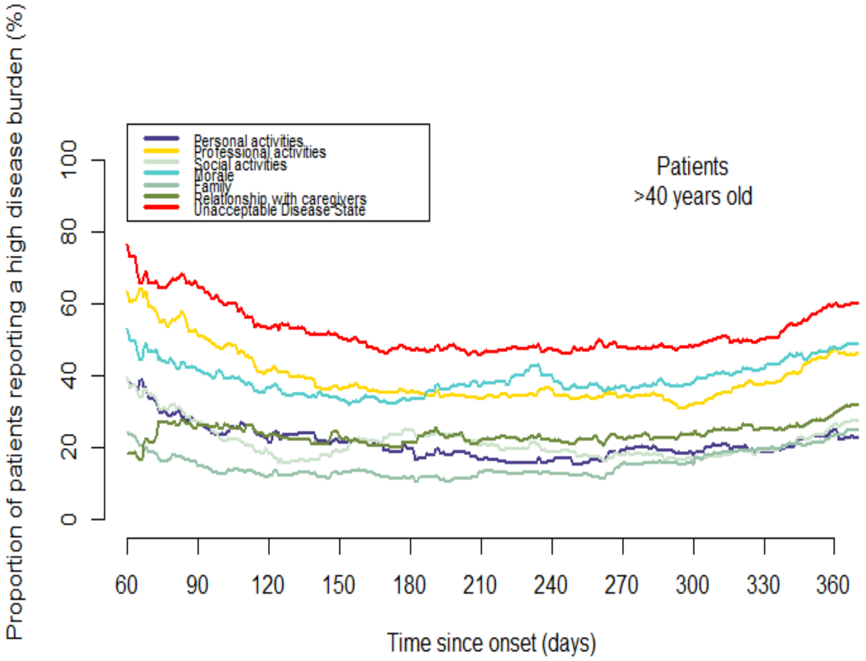

Supplement: Supplementary file 1 — Supplementary Information [file 41467_2022_29513_MOESM1_ESM.pdf]
