# Supplementary material for: Course of post COVID-19 disease symptoms over time in the ComPaRe long COVID prospective e-cohort
Source: Nat Commun. 2022 Apr 5;13:1812. doi: 10.1038/s41467-022-29513-z (PMC8983754; doi:10.1038/s41467-022-29513-z)
Supplement: Supplementary file 3 — Description of Additional Supplementary Information [file 41467_2022_29513_MOESM3_ESM.pdf]

Inventory of supporting information for “Course of post COVID-19 disease symptoms over time in the ComPaRe long COVID prospective e-cohort”

- Supplementary materials
- STROBE Checklist
