# Supplementary material for: Course of post COVID-19 disease symptoms over time in the ComPaRe long COVID prospective e-cohort
Source: Nat Commun. 2022 Apr 5;13:1812. doi: 10.1038/s41467-022-29513-z (PMC8983754; doi:10.1038/s41467-022-29513-z)
Supplement: Supplementary file 4 — Reporting Summary [file 41467_2022_29513_MOESM4_ESM.pdf]

nature portfolio

Corresponding author(s): Viet-Thi Tran

Last updated by author(s): 25/01/2022

Reporting Summary

Nature Portfolio wishes to improve the reproducibility of the work that we publish. This form provides structure for consistency and transparency in reporting. For further information on Nature Portfolio policies, see our Editorial Policies and the Editorial Policy Checklist.

Statistics

For all statistical analyses, confirm that the following items are present in the figure legend, table legend, main text, or Methods section.

n/a	Confirmed	
☒	☐	The exact sample size (*n*) for each experimental group/condition, given as a discrete number and unit of measurement
☐	☒	A statement on whether measurements were taken from distinct samples or whether the same sample was measured repeatedly
☒	☐	The statistical test(s) used AND whether they are one- or two-sided<br>*Only common tests should be described solely by name; describe more complex techniques in the Methods section.*
☐	☒	A description of all covariates tested
☐	☒	A description of any assumptions or corrections, such as tests of normality and adjustment for multiple comparisons
☐	☒	A full description of the statistical parameters including central tendency (e.g. means) or other basic estimates (e.g. regression coefficient) AND variation (e.g. standard deviation) or associated estimates of uncertainty (e.g. confidence intervals)
☒	☐	For null hypothesis testing, the test statistic (e.g. *F*, *t*, *r*) with confidence intervals, effect sizes, degrees of freedom and *P* value noted<br>*Give P values as exact values whenever suitable.*
☒	☐	For Bayesian analysis, information on the choice of priors and Markov chain Monte Carlo settings
☒	☐	For hierarchical and complex designs, identification of the appropriate level for tests and full reporting of outcomes
☒	☐	Estimates of effect sizes (e.g. Cohen's *d*, Pearson's *r*), indicating how they were calculated

Our web collection on statistics for biologists contains articles on many of the points above.

Software and code

Policy information about availability of computer code

Data collection	Data were collected on the ComPaRe platform (https://compare.aphp.fr). Dataset is available for academic research teams, under the conditions detailed on https://compare.aphp.fr. For statistical weighting, we used the data from the data from the Office of National Statistics in the United Kingdom (September 2021 data)
Data analysis	Statistical analyses were performed with R software (http://www.R-project.org, the R Foundation for Statistical Computing, Vienna, Austria), version 4.0.5 and the msm package. Statistical code is available upon request

For manuscripts utilizing custom algorithms or software that are central to the research but not yet described in published literature, software must be made available to editors and reviewers. We strongly encourage code deposition in a community repository (e.g. GitHub). See the Nature Portfolio guidelines for submitting code & software for further information.

Data

Policy information about availability of data

All manuscripts must include a data availability statement. This statement should provide the following information, where applicable:

- Accession codes, unique identifiers, or web links for publicly available datasets
- A description of any restrictions on data availability
- For clinical datasets or third party data, please ensure that the statement adheres to our policy

All data generated and used in the study are from the ComPaRe e-cohort platform. The data are available under the specific rules of the cohort. Access can be obtained under the conditions detailed on https://compare.aphp.fr. The processed data from Figure 2 and 3 are provided in the Source Data file.

Field-specific reporting

Please select the one below that is the best fit for your research. If you are not sure, read the appropriate sections before making your selection.

☒ Life sciences ☐ Behavioural & social sciences ☐ Ecological, evolutionary & environmental sciences

For a reference copy of the document with all sections, see nature.com/documents/nr-reporting-summary-flat.pdf

Life sciences study design

All studies must disclose on these points even when the disclosure is negative.

Sample size	As the study was descriptive, all patients meeting inclusion criteria were included in the study.
Data exclusions	No data were excluded from analyses
Replication	NA, (this is descriptive study aimed at studying the prevalence of COVID-19 symptoms among patients with persistant symptoms)
Randomization	NA (not a comparative effectiveness study)
Blinding	NA (not a comparative effectiveness study)

Reporting for specific materials, systems and methods

We require information from authors about some types of materials, experimental systems and methods used in many studies. Here, indicate whether each material, system or method listed is relevant to your study. If you are not sure if a list item applies to your research, read the appropriate section before selecting a response.

Materials & experimental systems

n/a	Involved in the study	
☒	☐	Antibodies
☒	☐	Eukaryotic cell lines
☒	☐	Palaeontology and archaeology
☒	☐	Animals and other organisms
☐	☒	Human research participants
☒	☐	Clinical data
☒	☐	Dual use research of concern

Methods

n/a	Involved in the study	
☒	☐	ChIP-seq
☒	☐	Flow cytometry
☒	☐	MRI-based neuroimaging

Human research participants

Policy information about studies involving human research participants

Population characteristics	Patients' demographic characteristics:<br>age, biological sex, educational level (Middle school or equivalent; High school or equivalent; 2 years post-secondary education; ≥ 3 years post-secondary education; Other), comorbidities (from a list of 250 diseases adapted from the ICPC).<br><br>Information on the COVID-19 infection<br>Date of symptom onset; nature of the COVID-19 confirmation (RT-PCR or serological assay); Date of the first positive test; Hospitalization during the acute infection (Yes/No); Hospitalisation in ICU ? (Yes/No); length of hospital stay
Recruitment	Participants were recruited via a general media (newspaper/radio) and social media campaign. A call for participation was issued on TousAntiCovid, the French national contact tracing app.<br>This poses a potential self-selection bias. To minimize this problem, we used a weighted dataset obtained by calibration on margins with weights for age (<24,25-34,35-49,50-69,≥70 years old), gender and hospitalization during the acute phase of the disease derived from the data from the Office of National Statistics in the United Kingdom (September 2021 data).<br>Weights were truncated at the 99th percentile.
Ethics oversight	The Institutional Review Board of Hôtel-Dieu Hospital, Paris, approved the study (IRB: 0008367). All patients provided electronic consent before participating in the study.

Note that full information on the approval of the study protocol must also be provided in the manuscript.
